# ADAM-10 Is Involved in Osteoblast Mineralization and Rheumatoid Arthritis-Associated Bone Destruction

**DOI:** 10.3390/ijms27219842

**Published:** 2026-11-03

**Authors:** Haruka Nakamura, Hiroyuki Kojima, Kohei Maeda, Kosuke Sakurai, Toshihiro Tanioka, Sho Ishii, Kuninobu Wakabayashi, Masahiro Hosonuma, Yuki Azetsu, Masayuki Iyoda, Masamichi Takami, Takeo Isozaki

**Affiliations:** 1 Department of Pathogenesis and Translational Medicine, Showa Medical University Graduate School of Pharmacy, Tokyo 142-8555, Japan; gp23-h017@pharm.showa-u.ac.jp (H.N.); gp23-h010@pharm.showa-u.ac.jp (H.K.); k.maeda@pharm.showa-u.ac.jp (K.M.); kosuke-39@cmed.showa-u.ac.jp (K.S.); tanioka@pharm.showa-u.ac.jp (T.T.); 2 Division of Rheumatology, Department of Medicine, Showa Medical University, Tokyo 142-8555, Japan; shoishii@med.showa-u.ac.jp (S.I.); kuninobu@med.showa-u.ac.jp (K.W.); 3 Division of Medical Pharmacology, Department of Pharmacology, Showa Medical University School of Medicine, Tokyo 142-8555, Japan; masa-hero@med.showa-u.ac.jp; 4 Pharmacological Research Center, Showa Medical University, Tokyo 142-8555, Japan; azetsu@dent.showa-u.ac.jp (Y.A.); takami@dent.showa-u.ac.jp (M.T.); 5 Department of Pharmacology, Graduate School of Dentistry, Showa Medical University, Tokyo 142-8555, Japan; 6 Department of Microbiology and Immunology, Showa Medical University Graduate School of Medicine, Tokyo 142-8555, Japan; iyoda@med.showa-u.ac.jp; 7 Division of Nephrology, Department of Medicine, Showa Medical University Graduate School of Medicine, Tokyo 142-8555, Japan

**Keywords:** rheumatoid arthritis, ADAM-10, CIA, bone destruction, inflammation, primary osteoblast, RA-HOB

## Abstract

Rheumatoid arthritis (RA), a chronic autoimmune condition, causes irreversible bone destruction. A disintegrin and metalloprotease 10 (ADAM-10) has been implicated in inflammatory processes in RA. We investigated the potential role of ADAM-10 in osteoblast mineralization and RA-associated bone destruction. Serum ADAM-10 levels in RA patients were measured by ELISA, and ADAM-10 expression in osteoblasts was assessed by Western blotting. Collagen-induced arthritis (CIA) mice were treated intraperitoneally with the ADAM-10 inhibitor GI254023X (100 mg/kg) for three weeks. Arthritis and bone destruction were evaluated by macroscopic, immunohistochemical, histological, and micro-CT analyses. The effects of ADAM-10 inhibition on primary osteoblast mineralization were also assessed by Alizarin Red S staining. Serum ADAM-10 levels in patients with RA tended to be higher at Stage III of the Steinbrocker classification. In CIA mice, ADAM-10 inhibition attenuated weight loss, paw swelling, and arthritis scores and suppressed bone destruction. ADAM-10 inhibition also significantly reduced *Rankl* expression in knee joints. Furthermore, the inhibition reversed the suppression of primary osteoblast mineralization induced by TNF-α. Pharmacological inhibition of ADAM-10 attenuated experimental arthritis and bone destruction and improved osteoblast mineralization, suggesting that ADAM-10 may contribute to inflammatory bone destruction in RA.

## 1. Introduction

Rheumatoid arthritis (RA) is a chronic autoimmune disease affecting approximately 1% of the population and is characterized by persistent synovial inflammation and progressive bone destruction [1]. The introduction of biological agents and Janus kinase (JAK) inhibitors has markedly improved disease control [2]. However, structural bone damage continues to progress in a subset of patients, leading to irreversible functional impairment. Further research into bone destruction may help improve our understanding of RA and identify potential therapeutic targets.

A disintegrin and metalloproteases (ADAMs) are a family of proteases that mediate the ectodomain shedding of cell-surface proteins [3,4]. ADAM-10 is expressed in a variety of cell types, including neurons, epithelial cells, and immune cells, and is known to regulate inflammation, cell adhesion, and cell differentiation by cleaving more than 100 membrane proteins [5,6,7,8].

We previously reported a weak but statistically significant positive correlation between serum ADAM-10 levels and the Disease Activity Score in 28 joints (DAS28) in patients with RA [9]. Although systemic inflammation contributes to joint destruction in RA, disease activity and structural joint damage represent distinct aspects of the disease. Therefore, the relationship between serum ADAM-10 levels and the extent of structural joint damage remains unclear.

We also previously demonstrated that ADAM-10 is highly expressed in RA synovial tissue and is involved in processes associated with inflammation and angiogenesis [10]. Furthermore, ADAM-10 has been implicated in the regulation of osteoclast differentiation and RANKL shedding [11]. These findings suggest that ADAM-10 may be involved in both inflammatory processes and bone metabolism. However, its role in osteoblasts, particularly under inflammatory conditions, remains poorly understood.

ADAM-10 has been reported to be expressed in osteoblasts [12]. Nevertheless, little is known about its contribution to osteoblast mineralization under inflammatory conditions or about the effects of pharmacological ADAM-10 inhibition on this process. Investigating these effects may provide further insight into the potential role of ADAM-10 in bone metabolism during inflammation.

In this study, we examined serum ADAM-10 levels in relation to Steinbrocker Stages I–IV in patients with RA. We also investigated ADAM-10 expression in human osteoblasts under inflammatory conditions and examined the effects of ADAM-10 inhibition on osteoblast mineralization. In parallel, we evaluated the effects of pharmacological ADAM-10 inhibition on arthritis progression and bone destruction in a collagen-induced arthritis (CIA) mouse model. Through these complementary approaches, we aimed to investigate the potential involvement of ADAM-10 in osteoblast mineralization and RA-associated bone destruction.

## 2. Results

### 2.1. ADAM-10 in RA Serum

**Figure 1:**
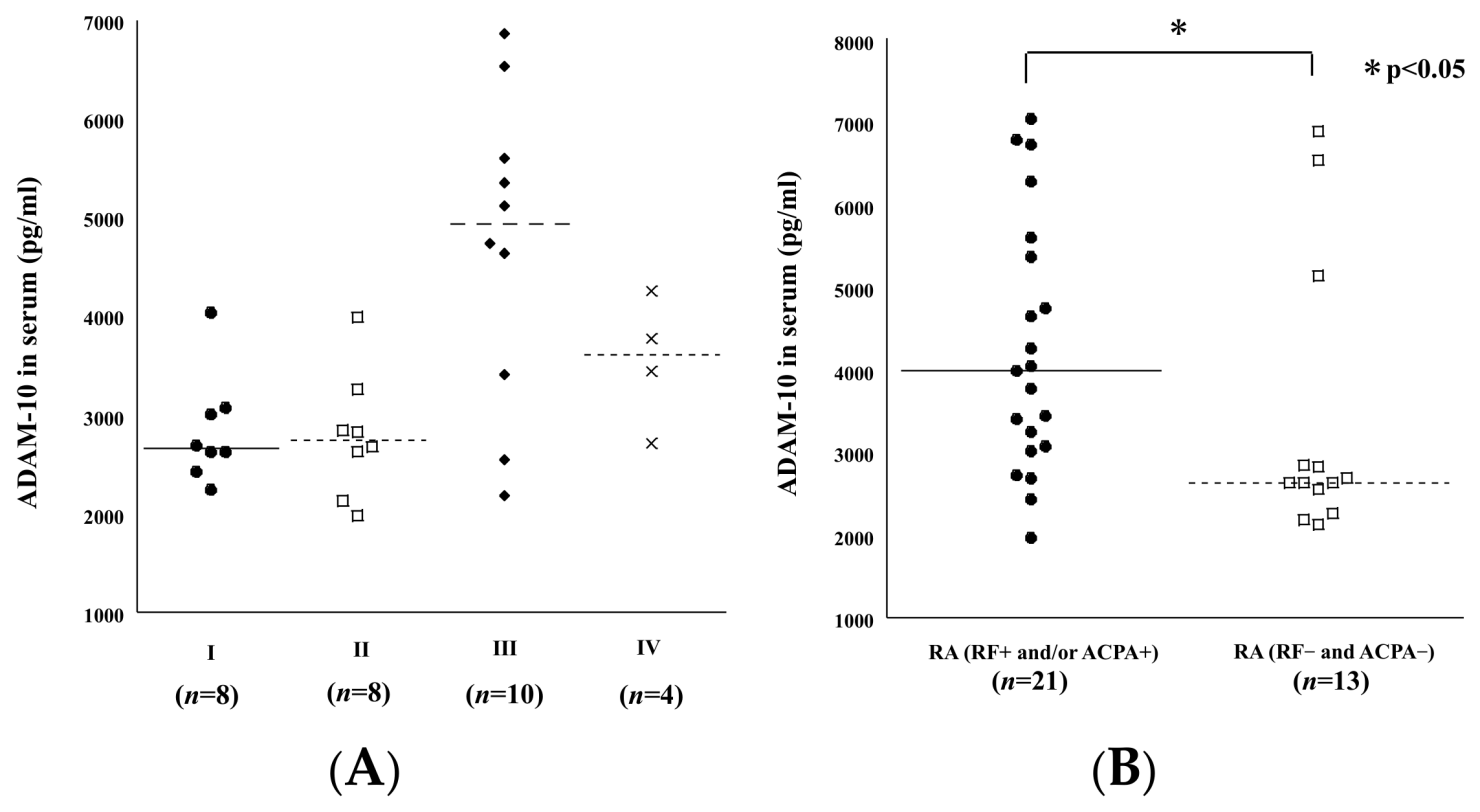
Serum ADAM-10 is associated with RA and bone destruction. (**A**) Serum ADAM-10 levels were higher in patients with Stage III than in those with other stages. There was no statistically significant difference (Kruskal–Wallis test). (**B**) Serum from patients with RA (RF+ and/or ACPA+) had higher ADAM-10 content than patients with RA (RF− and ACPA−). Statistical significance was determined using the Mann–Whitney U test (*n* = number of patients, Median [IQR], * *p* < 0.05). Abbreviations: ACPA, anti-citrullinated peptide antibodies; ADAM-10, a disintegrin and metalloprotease 10; RA, rheumatoid arthritis; RF, rheumatoid factor.

To investigate the relationship between a disintegrin and metalloprotease 10 (ADAM-10) and bone destruction in RA, the correlation between ADAM-10 and the Steinbrocker classification, a clinical indicator for assessing bone destruction, categorizing cases into Stages I through IV based on plain radiographs of joints in the hands or feet, was analyzed (Table S1). Stages I–IV are defined as the absence of cartilage or bone destruction, the onset of joint destruction, advanced bone destruction, and an ankylosed state, respectively. Data from five patients were excluded as outliers. Although there was no significant difference in ADAM-10 concentrations across the various stages of Steinbrocker (*p* = 0.371), Serum ADAM-10 levels were higher in patients with Steinbrocker Stage III than in those with the other stages (Median [IQR]: Stage I 2669 pg/mL [2489–3066 pg/mL, *n* = 8], Stage II 2757 pg/mL [2257–3159 pg/mL, *n* = 8], Stage III 4945 pg/mL [3200–5844 pg/mL, *n* = 10], Stage IV 3616 pg/mL [2905–4149 pg/mL, *n* = 4], Figure 1A). Furthermore, serum ADAM-10 levels varied considerably among patients with Steinbrocker Stage III RA. To investigate whether this variation was associated with other background factors, patients were classified into a low ADAM-10 group (*n* = 3) and a high ADAM-10 group (*n* = 7). Twenty clinical laboratory parameters were compared using Student’s *t*-test, with *p*-values adjusted using the Benjamini–Hochberg method. No significant differences remained after adjustment for multiple comparisons (Table S2). Serum ADAM-10 concentrations in the RA (rheumatoid factor (RF)+ and/or anti-citrullinated protein antibodies (ACPA)+) group were significantly higher than those in the RA (RF− and ACPA−) group (Median (IQR): 3997 (3051–5489, *n* = 21), 2638 (2403–3999, *n* = 13), *p* < 0.05, respectively, Figure 1B).

### 2.2. ADAM-10 in RA-Human Osteoblasts (RA-HOB)

**Figure 2:**
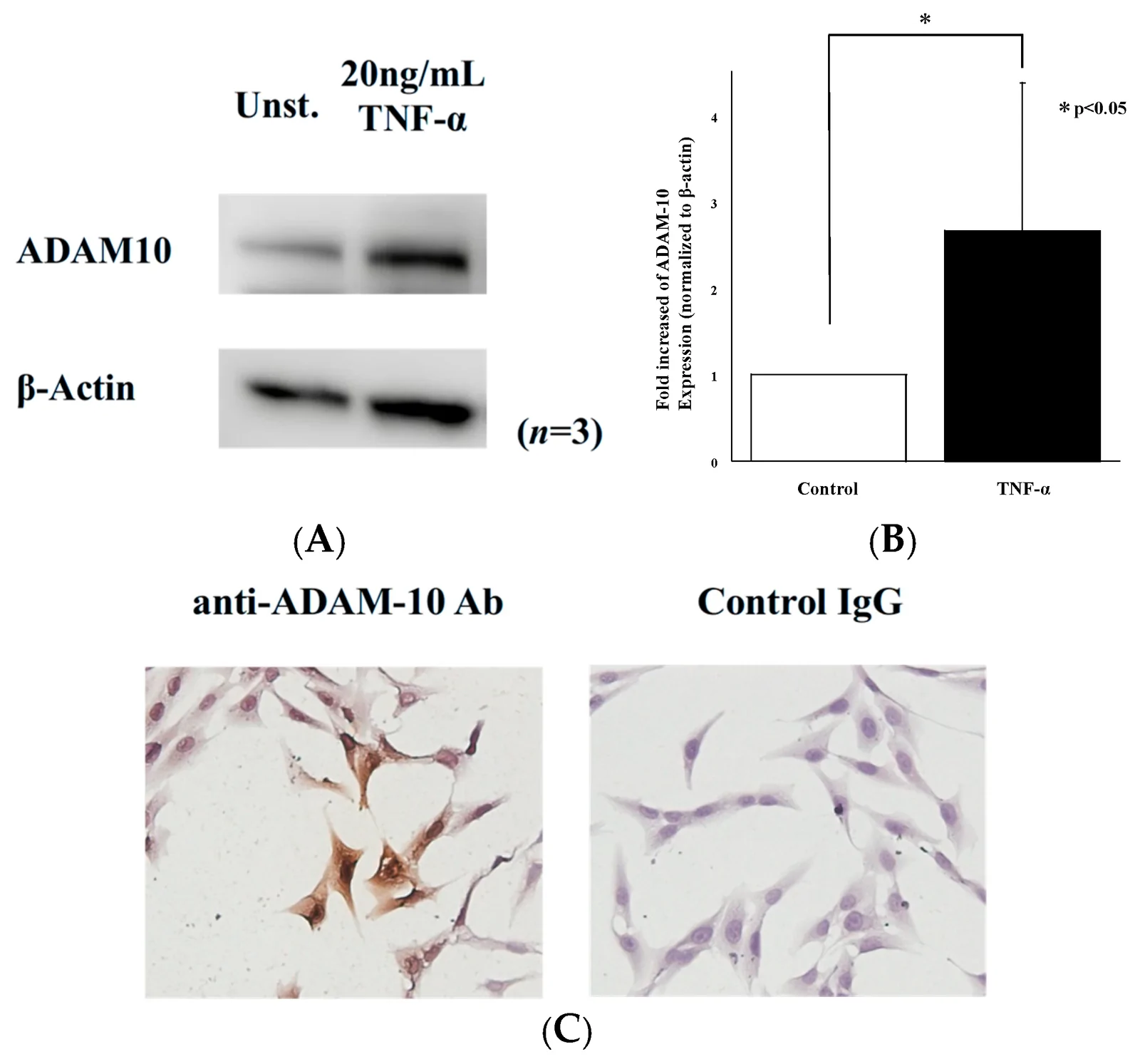
Inflammatory stimulation upregulates ADAM-10 expression in osteoblasts. (**A**) TNF-α stimulation significantly increased the expression of ADAM-10 in RA-HOB cells (*n* = number of patients). (**B**) Quantification of ADAM-10 expression. ADAM-10 signal was normalised to β-actin. Statistical analysis was performed using a paired *t*-test, * *p* < 0.05. (**C**) In the ADAM-10 immunostaining, the entire RA-HOB cells were stained. Abbreviations: Ab, antibody; ADAM-10, a disintegrin and metalloprotease 10; IgG, immunoglobulin G; RA-HOB, human osteoblasts-rheumatoid arthritis; TNF-α, tumor necrosis factor-alpha.

To examine ADAM-10 expression in human osteoblasts, Western blotting and immunostaining were performed. Western blot analysis showed increased ADAM-10 protein expression following stimulation with tumor necrosis factor-alpha (TNF-α) in RA-HOB (Figure 2A,B). Immunostaining after TNF-α stimulation confirmed the expression of ADAM-10 throughout the cytoplasm of RA-HOB cells (Figure 2C).

### 2.3. Effects of ADAM-10 Inhibition on the Macroscopic Features of CIA

To investigate the effects of ADAM-10 on joint destruction, ADAM-10 inhibitors were administered to collagen-induced arthritis (CIA) mice. Because three mice died immediately following the booster immunization on day 21, the total number of CIA mice used in the experiment was seven, of which four received ADAM-10 inhibitors and three were CIA control mice.

**Figure 3:**
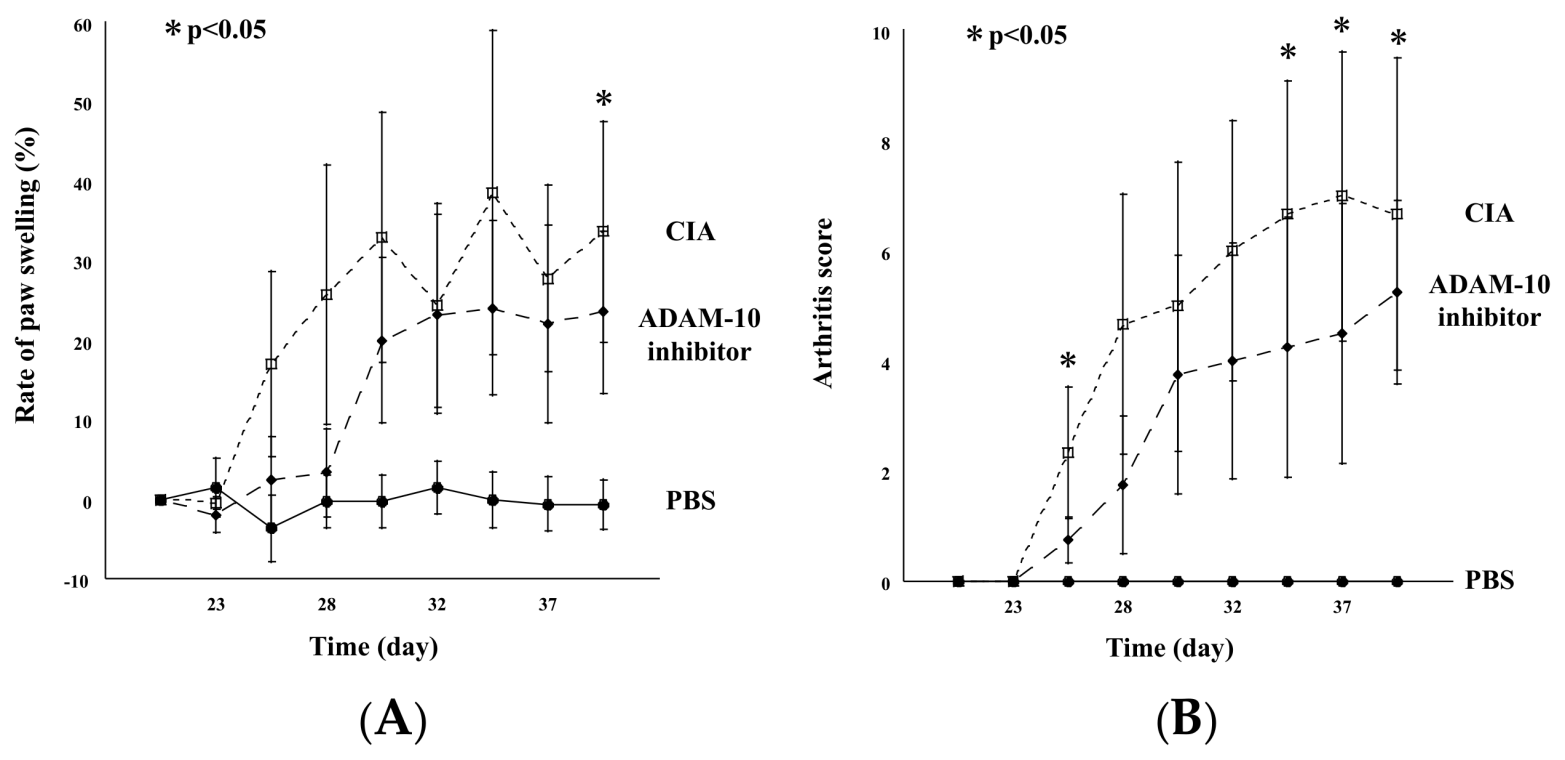

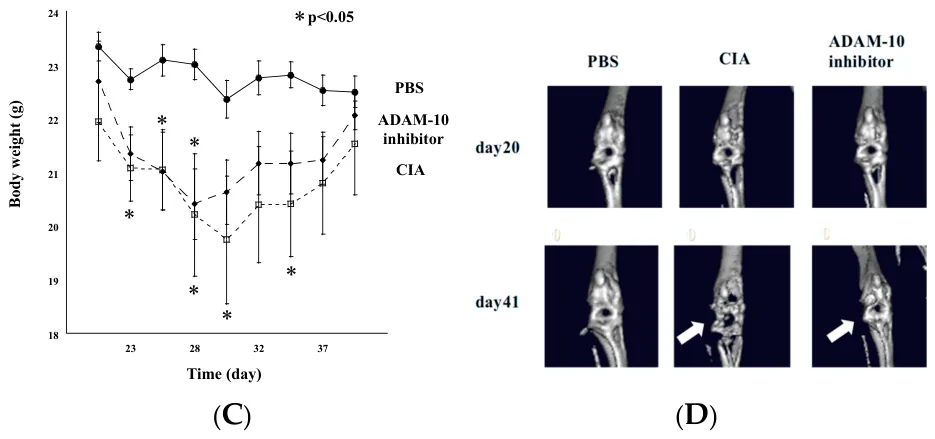
ADAM-10 contributes to arthritis progression in CIA mice. (**A**) The ADAM-10 inhibitor group showed a reduced rate of paw swelling compared with the CIA group. (**B**) The ADAM-10 inhibitor group showed a reduction in arthritis scores compared with the CIA group. (**C**) While body weight initially decreased, the ADAM-10 inhibitor group showed earlier signs of recovery. Error bars show standard deviation. Statistical significance was determined by one-way ANOVA followed by Tukey’s multiple-comparison test (* *p* < 0.05 vs. PBS group). (**D**) Representative micro-CT images showed less bone destruction in the ADAM-10 inhibitor group (white arrows). Abbreviations: ANOVA, analysis of variance; ADAM-10, a disintegrin and metalloprotease 10; CIA, type II collagen (CII)-induced arthritis; micro-CT, micro-computed tomography; PBS, Phosphate-Buffered Saline.

Starting on day 23, the thickness of the CIA mice’s paws gradually increased, peaking by day 40. Compared to the CIA control group, the ADAM-10 inhibitor group showed a tendency towards a lower rate of change in paw thickness. At Day 41, a significant difference was observed between the phosphate-buffered saline (PBS) (*n* = 5) and CIA control (*n* = 3) groups, while no significant difference was observed between the PBS group and the ADAM-10 inhibitor group (*n* = 4). The ADAM-10 inhibitor tended to suppress swelling (Day 41; −0.7 ± 3.4%, 33.7 ± 17.0%, 23.6 ± 11.8%, *p* < 0.05, respectively, Figure 3A). Additionally, an increase of 10% or more was observed in the CIA control group starting on day 23, while in the ADAM-10 inhibitor group, it began on day 28, indicating a tendency to delay the onset of swelling.

The arthritis scores in the ADAM-10 inhibitor group were lower than those in the CIA control group, indicating a tendency to suppress inflammation (Day 41; 0 ± 0, 6.7 ± 3.5, 5.3 ± 1.9, *p* < 0.05, respectively, Figure 3B).

Furthermore, body weight was measured to assess systemic inflammation. Overall, the ADAM-10 inhibitor group showed a trend toward recovery at an earlier stage compared to the CIA control group (Day 30; 22.4 ± 0.4 g, 19.7 ± 1.5 g, 20.6 ± 0.7 g, *p* < 0.05, respectively; Figure 3C).

### 2.4. Effects of ADAM-10 Inhibition on Bone Destruction

A qualitative evaluation was performed using the representative image shown in Figure 3D. Micro-computed tomography (micro-CT) images revealed apparent bone destruction in the knee joints of CIA mice compared with those of normal mice. The extent of bone destruction appeared to be lower in the group treated with ADAM-10 inhibitors than in the CIA group (Figure 3D).

### 2.5. Histopathological Examination

**Figure 4:**
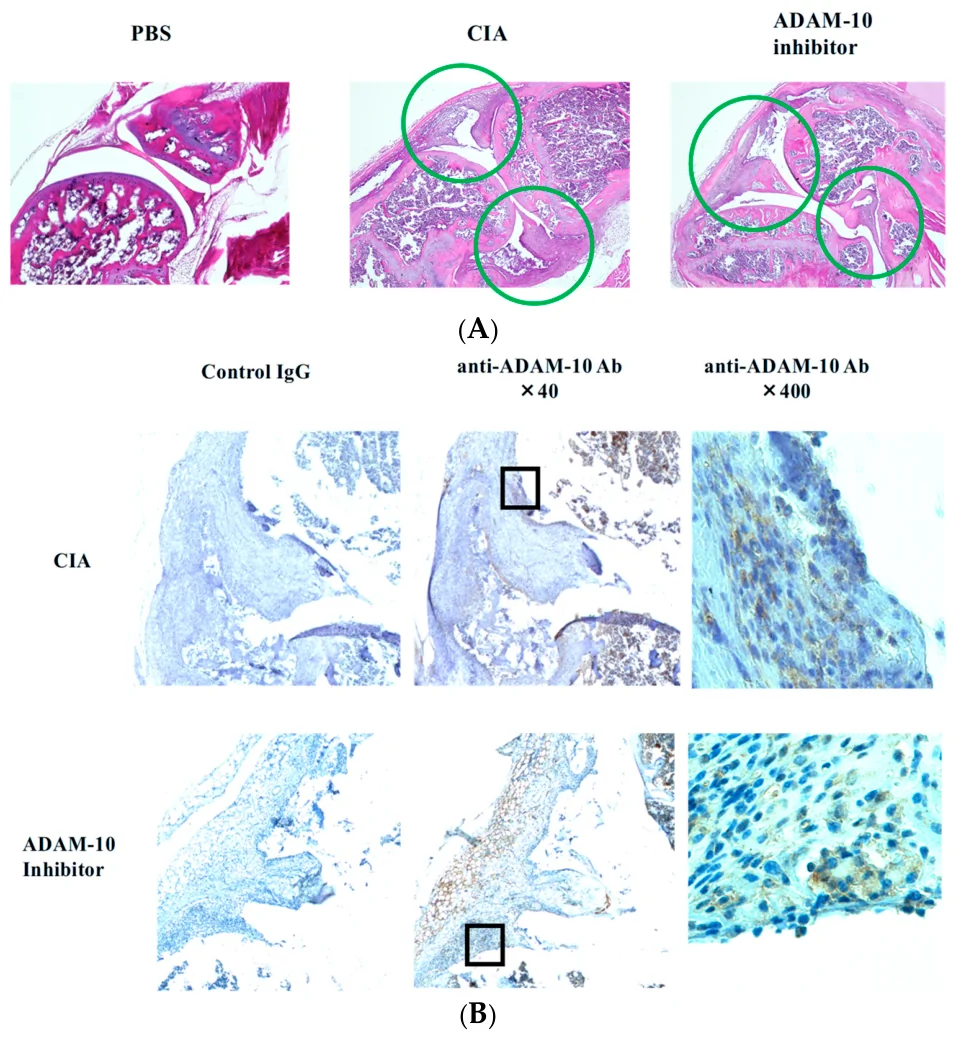
ADAM-10 is expressed in the synovium of mice. (**A**) HE staining showing synovial proliferation and inflammatory cell infiltration with bone erosion in the CIA group (green circle). (**B**) Immunohistochemical staining demonstrating ADAM-10 expression in the synovial tissue of mice. The black box shows an enlarged section of the figure on the right. Abbreviations: ADAM-10, a disintegrin and metalloprotease 10; CIA, type II collagen (CII)-induced arthritis; IgG, immunoglobulin G; HE staining, Hematoxylin and Eosin staining; PBS, Phosphate-Buffered Saline.

Representative hematoxylin and eosin (HE) staining of the knee joints of CIA mice showed synovial proliferation and bone infiltration in the CIA group, whereas these changes appeared less prominent in the ADAM-10 inhibitor group (Figure 4A). In immunostaining, ADAM-10 expression was confirmed in the synovial tissue of mice in both the CIA and ADAM-10 inhibitor groups (Figure 4B).

### 2.6. Effects of ADAM-10 Inhibition on Cytokine Expression in the Knee Joints of CIA Mice

**Figure 5:**
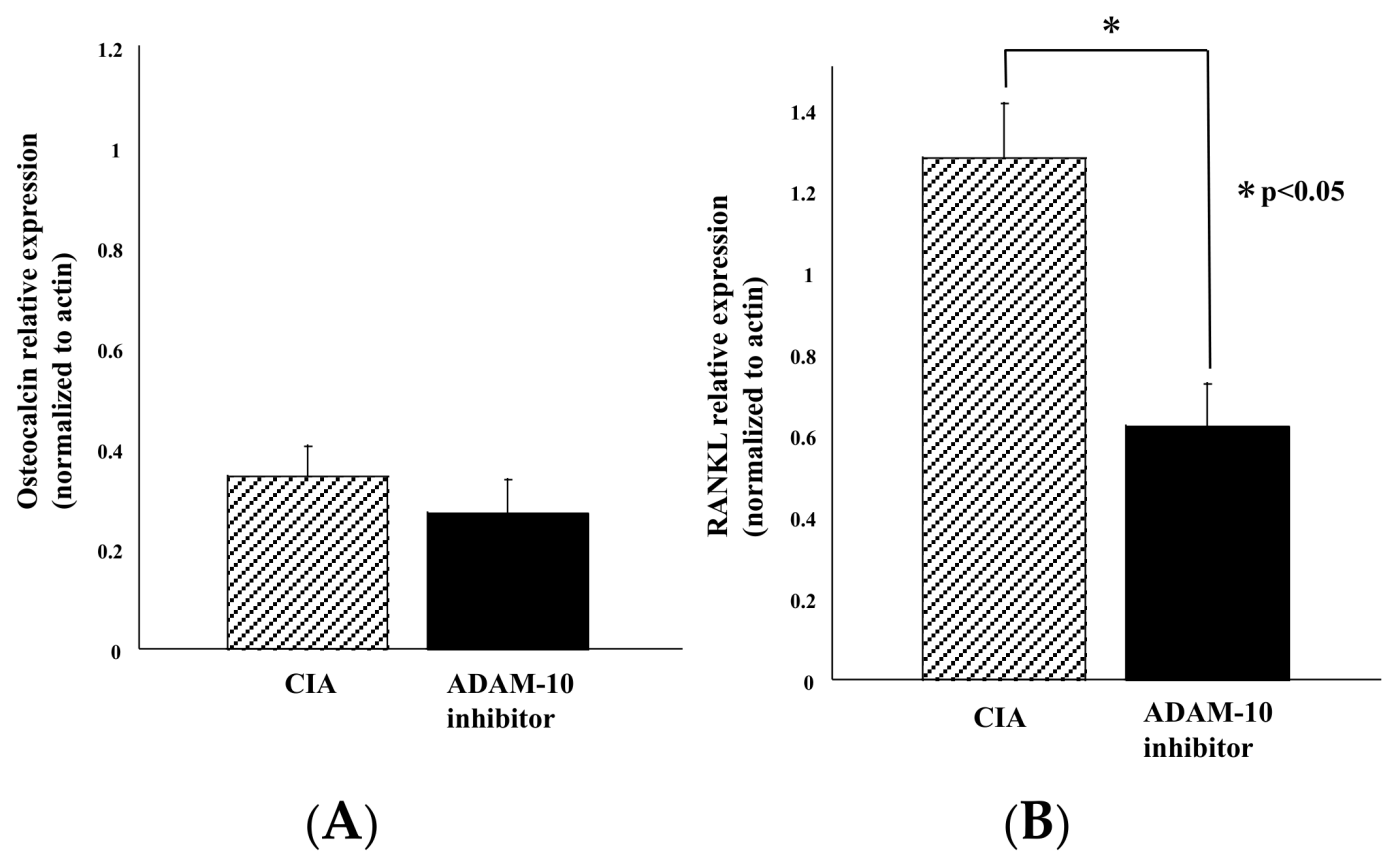

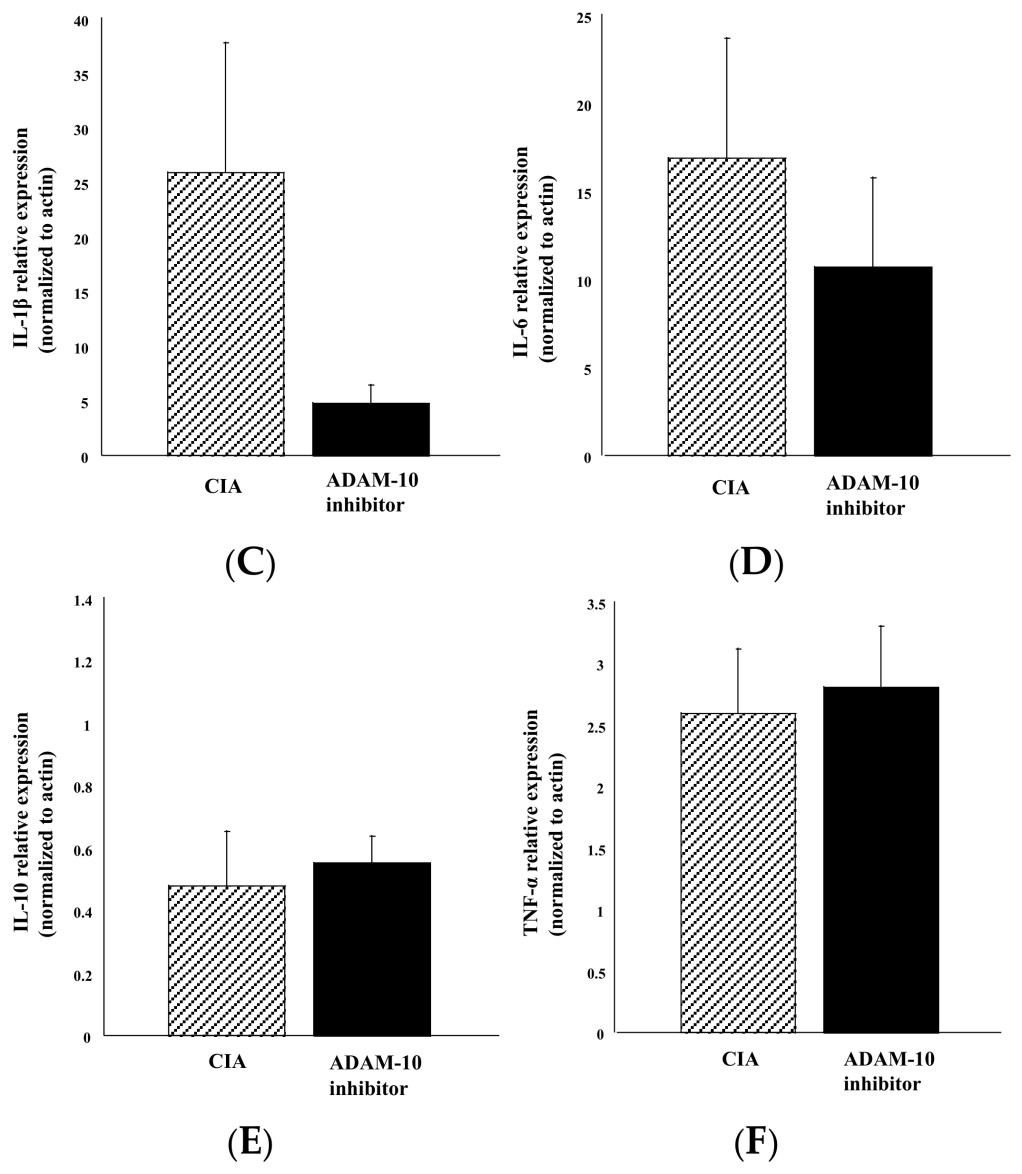
ADAM-10 promotes *Rankl* expression in the knee joint of mice. Gene expression of (**A**) *Osteocalcin*, (**B**) *Rankl*, (**C**) *Il1b*, (**D**) *Il6*, (**E**) *Il10*, and (**F**) *Tnf*. Inhibition of ADAM-10 significantly suppressed *Rankl* expression. On the other hand, no significant differences were observed for the other genes. An unpaired *t*-test was used. * *p* < 0.05. Abbreviations: ADAM-10, a disintegrin and metalloprotease 10; CIA, type II collagen (CII)-induced arthritis.

To evaluate changes in inflammatory cytokines and bone metabolism factors, the entire knee joints of mice were homogenized and mRNA expression of *Il1b*, *Il6*, *Tnf*, *Il10*, *Osteocalcin*, and *Rankl* was analyzed. Treatment with the ADAM-10 inhibitor significantly reduced *Rankl* expression compared with the CIA group (*Rankl*; 1.3 ± 0.1 vs. 0.6 ±0.1, Figure 5B). For the other genes, there were no significant differences between the CIA group and the ADAM-10 inhibitor group (Figure 5A,C–F).

### 2.7. Effects of ADAM-10 on the Mineralization of Primary Cultured Osteoblasts

**Figure 6:**
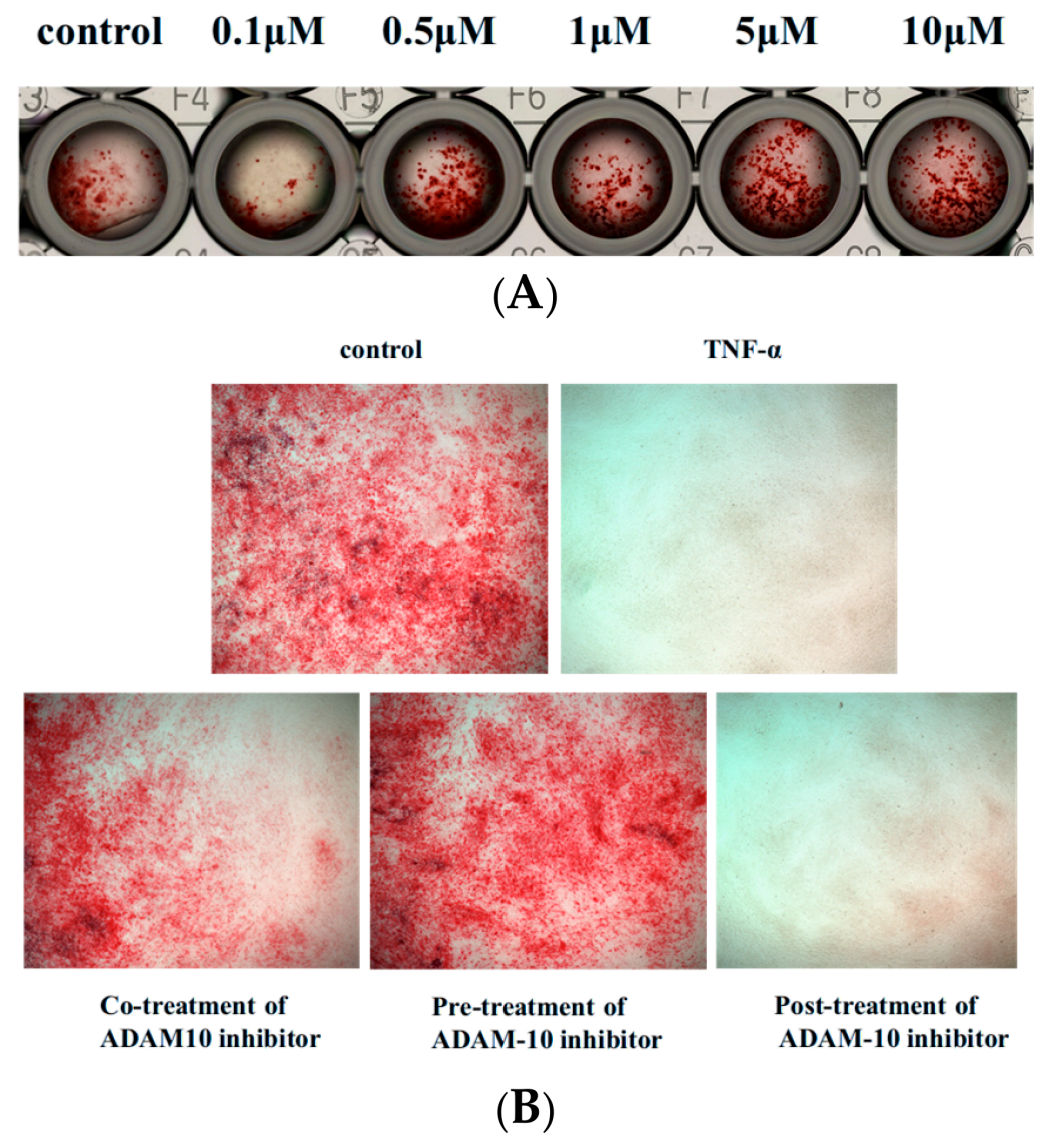

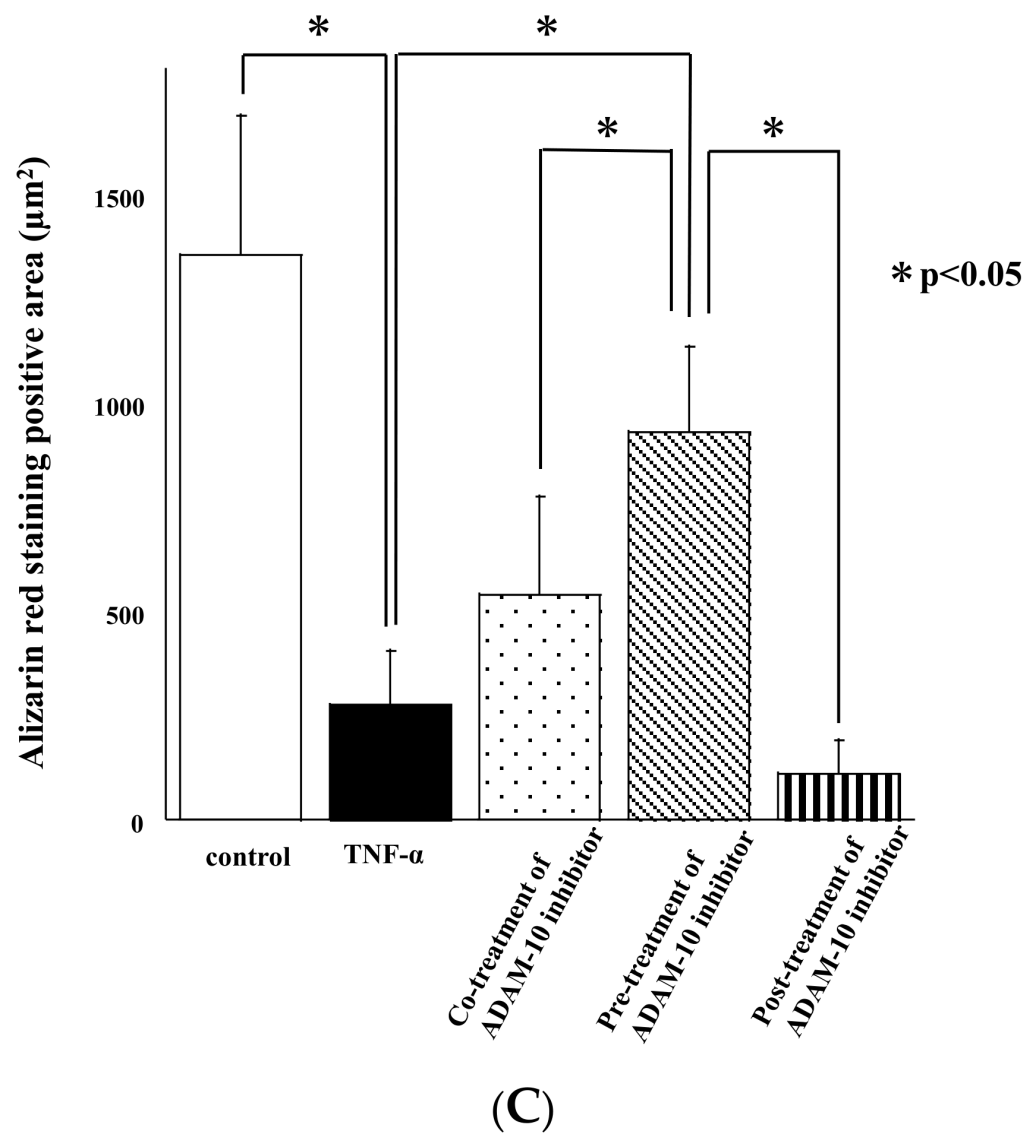
ADAM-10 negatively regulates osteoblast mineralization. (**A**) Treatment with the ADAM-10 inhibitor at a concentration of 0.5 µM resulted in a greater amount of mineralized tissue stained with Alizarin Red S than treatment with 0.1 µM. (**B**) When the inhibitor was administered prior to TNF-α stimulation (inhibitor pre-treatment), mineralization was observed. (**C**) In the ADAM-10 inhibitor pre-treatment group, a significantly greater increase in mineralization was observed compared with the co-treatment and post-treatment groups. Statistical significance was assessed using a paired *t*-test. * *p* < 0.05. Abbreviations: ADAM-10, a disintegrin and metalloprotease 10; TNF-α, necrosis factor-alpha.

To evaluate the effect of ADAM-10 on osteoblast mineralization, ADAM-10 inhibitor concentrations were set in steps ranging from 0.1 µM to 10 µM. Staining of osteoblasts with Alizarin Red S revealed that ADAM-10 inhibition promoted osteoblast mineralization across the tested concentrations (Figure 6A).

### 2.8. Effects of ADAM-10 on Osteoblasts

Prior studies have demonstrated that TNF-α-induced nuclear factor kappa B (NF-κB) activation suppresses bone formation in mouse-derived osteoblasts [13]. Therefore, to investigate the effect of ADAM-10 inhibitors on the suppression of osteoblast mineralization induced by TNF-α stimulation, Alizarin Red S staining was conducted using primary cultured mouse osteoblasts. Mineralization was observed in the group treated with the ADAM-10 inhibitor prior to TNF-α stimulation. In contrast, sufficient mineralization was not observed in the groups treated with the ADAM-10 inhibitor simultaneously with or following TNF-α stimulation. These results suggest that ADAM-10 inhibition may act preventively against the reduction in osteogenic capacity caused by TNF-α (Figure 6B,C).

## 3. Discussion

RA is an autoimmune disease that causes irreversible bone destruction due to inflammation of the synovial membrane. As RA progresses, patients can develop physical disability and systemic complications, resulting in a reduced quality of life and increased mortality [1,14,15].

In the present study, we focused on bone destruction and used the Steinbrocker classification, an indicator of irreversible joint destruction. Although the overall differences among the four stages were not statistically significant, serum ADAM-10 levels were relatively high in Stage III. This suggests that ADAM-10 is involved in the progression of RA. Furthermore, because ADAM-10 concentrations varied in Stage III, we examined the other laboratory parameters; however, none were found to make a significant contribution.

Stage III is characterized by marked joint damage and may be accompanied by ongoing inflammatory and destructive processes, whereas Stage IV represents advanced structural damage, including joint deformity and ankylosis. The relatively lower serum ADAM-10 levels observed in Stage IV compared with Stage III may reflect differences in inflammatory activity between these stages. However, this interpretation remains speculative, as inflammatory activity was not directly assessed in relation to the stage-specific differences in serum ADAM-10 levels. 

In addition, serum ADAM-10 levels were significantly higher in patients with seropositive RA (RF+ and/or ACPA+) than in patients who were negative for both RF and ACPA. This finding suggests a possible association between serum ADAM-10 levels and the serological features of RA.

In the CIA mouse model, GI254023X treatment significantly reduced RANKL mRNA expression in the knee joints compared with the CIA control group. A previous clinical study reported that successful treatment with disease-modifying antirheumatic drugs (DMARDs) was associated with decreased RANKL expression and increased osteoprotegerin expression in the synovial tissue of patients with RA, and that these changes correlated with a reduction in radiographic erosion scores of the hands and feet [16]. In the present study, administration of the ADAM-10 inhibitor GI254023X reduced *Rankl* expression in the knee joints of CIA mice and was accompanied by a tendency towards reduced joint swelling and qualitatively less prominent bone destruction on micro-CT images. Taken together, these findings raise the possibility that ADAM-10-associated pathways are involved in the regulation of *Rankl* expression and may contribute to joint inflammation and bone destruction in RA. However, because gene expression was assessed in whole knee joint tissue, the cellular source of the observed change could not be determined. Furthermore, the systemic administration of GI254023X does not allow us to distinguish the contributions of ADAM-10 inhibition in osteoblasts from those in synovial cells and other cell populations.

ADAM-10 is known to regulate inflammatory responses and cell–cell interactions by controlling the shedding of cell surface molecules [3]. In particular, its association with inflammatory cytokines such as TNF-α and IL-6 has been reported, and elevated ADAM-10 expression has been observed in synovial tissue from patients with RA [6]. In the present study, ADAM-10 inhibition did not significantly alter the gene expression of *Tnf* or *Il6*. One possible explanation for this result is that the entire knee joint was homogenized. In other words, because the synovial membrane, bone, cartilage, and muscle tissue were all intermingled, localized changes in individual tissues may have been masked.

In vitro, pretreatment with GI254023X preserved osteoblast mineralization under TNF-α stimulation. These findings raise the possibility that ADAM-10 inhibition may influence osteoblast responses under inflammatory conditions.

Although GI254023X preferentially inhibits ADAM-10 over ADAM-17, it can also inhibit other metalloproteinases, including MMP-1, MMP-3, MMP-9, and MMP-13, with particularly high potency against MMP-9 and MMP-13 [17]. Thus, the effects observed in the present study may reflect the inhibition of ADAM-10 and potentially other metalloproteinases. Nevertheless, our findings provide evidence that GI254023X-sensitive pathways may be involved in the processes associated with RA-related bone destruction. Further studies using complementary approaches, such as genetic inhibition or more selective ADAM-10 inhibitors, would help clarify the specific contribution of ADAM-10 to these processes.

ADAM-10 is known to be involved in Notch signaling activation [3,18]. Notch signaling regulates osteoblast lineage commitment, differentiation, and maturation [12,19,20,21,22]. Based on the above, the observation that osteogenic potential differed depending on the timing of ADAM-10 inhibitor administration indicates that Notch signaling may play a role in the differentiation of mesenchymal cells into osteoblasts during the early stages of osteoblast differentiation.

This study has several limitations. These include the small number of patients in the clinical trial and the small sample size in the animal experiments. In particular, three animals died immediately following the booster immunization in the animal experiments, limiting the study’s statistical power. Additionally, there is a lack of quantitative micro-CT and histological analyses, a lack of evaluation of ADAM-10 expression levels and enzymatic activity by immunohistochemistry, the absence of mechanistic studies on Notch signaling, the use of pharmacological inhibition only, and a lack of genetic validation. The present study does not establish a direct causal relationship between ADAM-10 in osteoblasts and bone destruction in vivo, nor does it demonstrate that the effect of systemic ADAM-10 inhibition on CIA-associated bone destruction is mediated specifically through osteoblasts. Therefore, the need for further studies to validate its therapeutic potential and underlying mechanisms.

## 4. Materials and Methods

### 4.1. Patients

Initial experiments were conducted on samples taken from 35 patients diagnosed with RA based on the 2010 American College of Rheumatology (ACR)-European League Against Rheumatism (EULAR) classification criteria at the Department of Rheumatology, Showa Medical University Hospital or Showa Medical University East Hospital between May 2017 and June 2018. 13 patients had seronegative RA, defined as negative for serum RF and ACPA. All specimens were collected only after obtaining informed consent from the subjects. Data points that were more than 2 standard deviations from the mean were considered outliers and excluded from the analysis.

### 4.2. Preparation of Human Osteoblasts

RA-derived human osteoblasts (RA-HOB) were purified from metaphyseal trabecular bones in the proximal femora collected from patients with RA (*n* = 3) during total hip arthroplasty. Briefly, after removing pieces of cortical bone, articular cartilage, and soft connective tissue, the fragments were cut into small pieces and incubated with DMEM containing 1% collagenase at 37 °C for 30 min, followed by extensive washing. The resultant bone explants were cultured in tissue culture plates in DMEM containing 10% FBS. When cell monolayers were confluent (after 3–5 weeks of culture), the explants were removed, and the cells were replated at a cell density of 1 × 10^5^/mL. The obtained cells exhibited a flattened polygonal shape with multiple spindlelegs and possessed characteristics of the osteoblast-like phenotype such as osteocalcin expression, bone alkaline phosphatase expression, and mineralization as determined by von Kossa staining.

### 4.3. Experimental Animals

Seven-week-old male DBA/1JmsS mice were purchased from Sankyo Lab Service Co., Ltd. (Tokyo, Japan) and maintained under specific pathogen-free conditions. Following one week of adaptive feeding, fifteen mice were randomly assigned to three groups. One group served as the control, while the remaining two were used as CIA models (procedure described below). On day 21, CIA mice were divided into the following two subgroups: the CIA control group and ADAM-10 inhibitor treatment group (100 mg/kg, MedChemExpress, Monmouth Junction, NJ, USA). The ADAM-10 inhibitor was diluted to 100 mg/kg in a solution of 10% DMSO, 40% PEG300, 5% Tween 80, and 45% saline; 200 µL was administered intraperitoneally five days a week from day 21 to day 41. The control group and CIA control group were administered the same amount of PBS on the same schedule. On day 42, the mice were anesthetized with 2.5% isoflurane and then subjected to cervical dislocation. Knee joints were harvested for histological and immunohistochemical analysis. All experiments were carried out in accordance with relevant guidelines and regulations. All efforts were made to minimize animal suffering and reduce the number of experimental animals used. Animals were allocated to the control and treatment groups by cage. No formal randomization procedure was used. No primary outcome measure was used to determine the sample size. All animals were treated at the same time of day, and paw thickness was measured in the same order at each assessment. Cage positions were changed during the experimental period to minimize potential effects of cage location. All measurements were performed by the same researcher. No blinding was performed.

### 4.4. Induction of the CIA Model

Bovine type II collagen (Chondrex, Inc., Woodinville, WA, USA) was dissolved at 2 mg/mL in 0.05 M acetic acid. Equal volumes of the bovine type II collagen solution and adjuvant were mixed and emulsified. The emulsion (Bovine type II collagen 100 mg/kg) was maintained at 4 °C and injected within 1 h. Arthritis was induced by an intradermal injection (0.1 mL) at the base of the mouse tail. Complete adjuvant (Chondrex, Inc.) was used for the initial immunization on day 0, and incomplete adjuvant (Chondrex, Inc.) was used for the booster on day21. The incidence rate of arthritis is 90–100%.

### 4.5. Assessment of Body Weight, Paw Swelling and Arthritis Score

Throughout the course of the experiment, body weight, thickness of limbs, and arthritis score were measured every 2–3 days. Additionally, CT scans of the left and right hind limbs were conducted every 7 days. The increase in paw swelling was calculated using the following formula: rate of paw swelling change (%) = [Paw thickness on the measurement date] − [Paw thickness before the onset of arthritis]/[Paw thickness before the onset of arthritis] × 100 [23]. Arthritis was evaluated on a scale of 0–4, as follows: 0: Normal foot; 1: One or two swollen toes, with no swelling of paw or ankle. 2: Three or more swollen toes, or mild swelling of the paw or ankle. 3: Swelling of the paw or ankle. Score 4: Severe swelling of the toes, paw, or ankle, or loss of function. The arthritis score was calculated as the total score for all four limbs, with a maximum of 16. A score of 4 or higher indicates that the model has been successfully established. The analysis included the PBS group (*n* = 5), the CIA group (*n* = 3), and the ADAM-10 inhibitor group (*n* = 4).

### 4.6. Micro-CT Examination

After sedating the mice with 2.5% isoflurane, they were placed in a lateral recumbent position, and CT imaging of the left and right hind limbs was conducted using the R_mCT2 micro-X-ray CT system (Rigaku, Tokyo, Japan). The CT scan parameters for the hind limbs were set to a voltage of 90 kV, a current of 160 µA, a field of view (FOV) of 60 mm, and a scan time of 4.5 min.

### 4.7. Histopathological Analysis of the Knee Joint

The left hind limb was immersed in 4% PFA for 24 h, washed with PBS, decalcified with 10% ethylenediaminetetraacetic acid (EDTA) over a period of 3–4 weeks, and embedded in paraffin. Paraffin sections were prepared at a thickness of 10 µm; the paraffin was removed using xylene and ethanol, followed by HE staining and immunohistochemical staining.

### 4.8. Immunohistochemistry

#### 4.8.1. Immunohistochemistry of RA-HOB

RA-HOBs were grown to near-confluence in an 8-well LabTech chamber slide, and subsequently stimulated with cytokines for 24 h. After fixing in cold acetone, the sections were treated with 3% H_2_O_2_ in PBS for 5 min, and then with 5% goat serum and 20% fetal bovine serum (FBS) in PBS for 1 h. The sections were incubated with polyclonal rabbit anti-ADAM-10 Ab or with pre-immune rabbit IgG. Biotinylated goat anti-rabbit IgG (Vector Laboratories, Burlingame, CA, USA) was used as the secondary antibody, after which Vectastain ABC HRP reagent (Vector Laboratories) was added. DAB peroxidase (HRP) was used for visualization. The sections were counterstained with hematoxylin.

#### 4.8.2. Immunohistochemistry of Mouse Knee Joints

Paraffin-removed sections were reactivated with 10 mM citrate buffer (pH 6.0) and treated with 3% hydrogen peroxide to inactivate endogenous peroxidase. The sections were blocked with a mixture of 20% FBS, 5% goat serum, and 75% PBS, then incubated with the primary antibody (rabbit anti-ADAM-10 IgG, Abcam, Waltham, MA, USA) overnight at 4 °C. The sections were then washed, and the secondary antibody (goat anti-rabbit IgG HRP, Agilent Technologies, Inc., Santa Clara, CA, USA) was added and incubated at room temperature for 30 min. Finally, 3,3′-diaminobenzidine (DAB) was added for color development, and the sections were counterstained with hematoxylin.

### 4.9. Western Blotting

To investigate how RA-related inflammatory stimuli affect ADAM-10 expression in osteoblasts, HOBs were stimulated with 20 ng/mL TNF-α. Whole-cell extracts of RA-HOB were subjected to 10% tris-glycine gradient gel electrophoresis (Novex, Carlsbad, CA, USA) and transferred to a nitrocellulose membrane (Schleicher & Schuell, Dassel, Germany). The membranes were blocked with 5% nonfat milk powder in tris-buffered saline tween (TBST) (50 mmol/L Tris, 0.15 M NaCl, and 0.05% Tween 20), incubated with polyclonal rabbit anti-ADAM-10 Ab in TBST for 3 h, washed with TBST, and then incubated with goat anti-rabbit IgG (Vector Laboratories, Burlingame, CA, USA) for 1 h. After extensive washing with TBST, a chemiluminescent substrate (ECL Western blot detection system, Amersham Biosciences, Little Chalfont, UK) was added and the membrane was subjected to autoradiography. β-actin was used as a loading control.

### 4.10. RNA Extraction and Quantitative Polymerase Chain Reaction (qPCR)

Total RNA was purified using RNAiso Plus reagent (TaKaRa Bio Inc., Tokyo, Japan). The first-strand cDNA was synthesized from 1 µg of total RNA using a High-Capacity cDNA Reverse Transcription kit (Applied Biosystems, Foster City, CA, USA). PCR reactions were conducted using TaqMan probes (Applied Biosystems) for Osteocalcin (Mm03413826_mH), IL-1β (Mm01336189_m1), IL-6 (Mm00446190_m1), IL-10 (Mm00439616_m1), TNF-α (Mm00443258_m1), RANKL (Mm00441906_m1), and β-actin (Mm00607939_s1) using the StepOnePlus real-time PCR system (Applied Biosystems). Results were normalized to the endogenous reference β-actin gene, and the relative quantity of each mRNA was calculated using the comparative Ct (threshold cycle) method.

### 4.11. Primary Mouse Osteoblasts Culture

Pregnant mice were prepared. In this experiment, we used two mother mice and collected cells from 14 pups. After euthanizing pups on day 0 by decapitation, we harvested their calvaria and used them in the experiment. Primary osteoblasts were obtained from the calvariae of mice using 0.1% collagenase and 0.2% dispase. They were cultured at 5 × 10^3^ cells/well in 96-well Collagen I (Col1)-coated plates (Corning Inc., Corning, NY, USA) in osteogenic medium, dissolved in 10 mM β-glycerophosphate (β-GP, Sigma-Aldrich Japan, Tokyo, Japan), 10 nM dexamethasone (Sigma-Aldrich), and 50 μg/mL ascorbic acid (AA, FUJIFILM Wako, Tokyo, Japan), 100 ng/mL recombinant human BMP-2 (R&D Systems, Inc., Minneapolis, MN, USA) in α-minimum essential medium (α-MEM, FUJIFILM Wako) supplemented with 10% fetal bovine serum (FBS, Sigma-Aldrich) and 100 units/mL Penicillin-Streptomycin Mixed Solution (NACALAI TESQUE, INC., Kyoto, Japan) for seven days. The culture medium was changed every four days. Mineralization was confirmed by Alizarin Red S staining. Alizarin Red S-positive areas were quantified based on color thresholding of microscopic images to identify stained calcified areas using the Analyzer ver. 2.0.1.274, included with the BZ-X1000 microscope (Keyence Corporation, Tokyo, Japan).

### 4.12. Statistical Analysis

Statistical analyses were performed using JMP (JMP^®^ Student Edition 19.0.1 [887995], SAS Institute Inc., Cary, NC, USA). The Kruskal–Wallis test was used to compare serum ADAM-10 levels among the four Steinbrocker stages. Serum ADAM-10 concentrations in RA (RF+ and/or ACPA+) and RA (RF− and ACPA−) patients were compared using the Mann–Whitney U test. For Western blot analysis, ADAM-10 signal intensity was compared between paired conditions using a paired *t*-test. For comparisons of paw swelling, arthritis scores, and body weight among the PBS, CIA, and ADAM-10 inhibitor groups at each observation time point, one-way analysis of variance (ANOVA) followed by Tukey’s multiple-comparison test was used. For mRNA expression levels in the knee joints, an unpaired *t*-test was used to compare the CIA and ADAM-10 inhibitor groups. For the area stained with Alizarin Red S, a paired *t*-test was used to evaluate the efficacy of the ADAM-10 inhibitor compared to the TNF-α treatment group. All statistical tests were two-sided, and *p* values < 0.05 were considered to be statistically significant. Data analyzed using parametric tests are presented as mean ± SD, whereas data analyzed using non-parametric tests are presented as median (IQR).

## 5. Conclusions

This study suggests that ADAM-10 may contribute to RA-associated bone destruction. In CIA mice, the ADAM-10 inhibitor treatment attenuated arthritis severity, reduced *Rankl* expression in knee joint tissue, and was associated with less apparent bone destruction. In vitro, the ADAM-10 inhibitor prevented the suppression of osteoblast mineralization caused by TNF-α. Further studies are required to clarify the underlying mechanisms and determine the therapeutic potential of ADAM-10 inhibition in RA.

## Data Availability

The original contributions presented in this study are included in the article/Supplementary Materials. Further inquiries can be directed to the corresponding author.

## Supplementary Materials

The following supporting information can be downloaded at: https://www.mdpi.com/article/10.3390/ijms27219842/s1.

## Abbreviations

The following abbreviations are used in this manuscript:

ACPA	anti-citrullinated protein antibodies
ADAM-10	a disintegrin and metalloprotease 10
ANOVA	analysis of variance
BMP-2	bone morphogenetic protein-2
BSA	bovine serum albumin
BUN	blood urea nitrogen
CIA	collagen-induced arthritis
Col1	collagen type I
Ct	threshold cycle
DAB	3,3′-diaminobenzidine
DAS28	Disease Activity Score 28
DMEM	Dulbecco’s Modified Eagle Medium
EDTA	ethylenediaminetetraacetic acid
eGFR	estimated glomerular filtration rate
ELISA	enzyme-linked immunosorbent assay
FBS	fetal bovine serum
HE	hematoxylin and eosin
IgG	immunoglobulin G
IL-1β	interleukin-1 beta
IL-6	interleukin-6
IQR	interquartile range
JAK	Janus kinase
micro-CT	micro-computed tomography
mRNA	messenger RNA
NF-κB	nuclear factor kappa B
PBS	phosphate-buffered saline
PFA	paraformaldehyde
qPCR	quantitative polymerase chain reaction
RA	rheumatoid arthritis
RA-HOB	rheumatoid arthritis-derived human osteoblasts
RANKL	receptor activator of nuclear factor-κB ligand
RF	rheumatoid factor
Runx2	runt-related transcription factor 2
TBST	Tris-buffered saline with Tween 20
TNF-α	tumor necrosis factor-alpha

## References

[1] Yamanaka H., Sugiyama N., Inoue E., Taniguchi A., Momohara S. (2014). Estimates of the prevalence of and current treatment practices for rheumatoid arthritis in Japan using reimbursement data from health insurance societies and the IORRA cohort (I). Mod. Rheumatol..

[2] Yamanaka H., Tanaka E., Nakajima A., Furuya T., Ikari K., Taniguchi A., Inoue E., Harigai M. (2020). A large observational cohort study of rheumatoid arthritis, IORRA: Providing context for today;s treatment options. Mod. Rheumatol..

[3] Edwards D.R., Handsley M.M., Pennington C.J. (2008). The ADAM metalloproteinases. Mol. Asp. Med..

[4] Rocks N., Paulissen G., El Hour M., Quesada F., Crahay C., Gueders M., Foidart J.-M., Noel A., Cataldo D. (2008). Emerging roles of ADAM and ADAMTS metalloproteinases in cancer. Biochimie.

[5] Shahid S., Ikeda A., Layana M.C., Bartlett J.D. (2022). ADAM10: Possible functions in enamel development. Front. Physiol..

[6] Rosenbaum D., Saftig P. (2024). New insights into the function and pathophysiology of the ectodomain sheddase A Disintegrin And Metalloproteinase 10 (ADAM-10). FEBS J..

[7] Dorta S., Alexandre-Silva V., Popolin C.P., de Sousa D.B., Grigoli M.M., Pelegrini L.N.C., Manzine P.R., Camins A., Marcello E., Endres K. (2024). ADAM10 isoforms: Optimizing usage of antibodies based on protein regulation, structural features, biological activity and clinical relevance to Alzheimer;s disease. Ageing Res. Rev..

[8] Zhang W., Fu J., Du J., Liu X., Cheng J., Wei C., Xu Y., Fu J. (2024). A disintegrin and metalloproteinase domain 10 expression inhibition by the small molecules adenosine, cordycepin and N6, N6-dimethyladenosine and immune regulation in malignant cancers. Front. Immunol..

[9] Isozaki T., Ishii S., Nishimi S., Nishimi A., Oguro N., Seki S., Miura Y., Miwa Y., Oh K., Toyoshima Y. (2015). A disintegrin and metalloprotease-10 is correlated with disease activity and mediates monocyte migration and adhesion in rheumatoid arthritis. Transl. Res..

[10] Isozaki T., Rabquer B.J., Ruth J.H., Haines G.K., Koch A.E. (2013). ADAM-10 is overexpressed in rheumatoid arthritis synovial tissue and mediates angiogenesis. Arthritis Rheum..

[11] Babendreyer A., Kieselhorst J., Rinkens C., Lyashenko A.M., Düsterhöft S., Jahr H., Craveiro R.B., Wolf M., Ludwig A. (2024). Downregulation of the metalloproteinases ADAM10 or ADAM17 promotes osteoclast differentiation. Cell Commun. Signal..

[12] Dallas D.J., Genever P.G., Patton A.J., Millichip M.I., McKie N., Skerry T.M. (1999). Localization of ADAM-10 and Notch receptors in bone. Bone.

[13] Zhu S., Chen W., Masson A., Li Y.P. (2024). Cell signaling and transcriptional regulation of osteoblast lineage commitment, differentiation, bone formation, and homeostasis. Cell Discov..

[14] Smolen J.S., Aletaha D., McInnes I.B. (2016). Rheumatoid arthritis. Lancet.

[15] Calabresi E., Petrelli F., Bonifacio A.F., Puxeddu I., Alunno A. (2018). One year in review 2018: Pathogenesis of rheumatoid arthritis. Clin. Exp. Rheumatol..

[16] Haynes D., Crotti T., Weedon H., Slavotinek J., Au V., Coleman M., Roberts-Thomson P.J., Ahern M., Smith M.D. (2008). Modulation of RANKL and osteoprotegerin expression in synovial tissue from patients with rheumatoid arthritis in response to disease-modifying antirheumatic drug treatment and correlation with radiologic outcome. Arthritis Rheum..

[17] Ludwig A., Hundhausen C., Lambert M.H., Broadway N., Andrews R.C., Bickett D.M., Leesnitzer M.A., Becherer J.D. (2005). Metalloproteinase inhibitors for the disintegrin-like metalloproteinases ADAM10 and ADAM17 that differentially block constitutive and phorbol ester-inducible shedding of cell surface molecules. Comb. Chem. High Throughput Screen..

[18] Saha N., Robev D., Himanen J.P., Nikolov D.B. (2019). ADAM proteases: Emerging role and targeting of the non-catalytic domains. Cancer Lett..

[19] Hilton M.J., Tu X., Wu X., Bai S., Zhao H., Kobayashi T., Kronenberg H.M., Teitelbaum S.L., Ross F.P., Kopan R. (2008). Notch signaling maintains bone marrow mesenchymal progenitors by suppressing osteoblast differentiation. Nat. Med..

[20] Tu X., Chen J., Lim J., Karner C.M., Lee S.-Y., Heisig J., Wiese C., Surendran K., Kopan R., Gessler M. (2012). Physiological Notch signaling maintains bone homeostasis via RBPjk and Hey upstream of NFATc1. PLoS Genet..

[21] Engin F., Yao Z., Yang T., Zhou G., Bertin T., Jiang M.M., Chen Y., Wang L., Zheng H., Sutton R.E. (2008). Dimorphic effects of Notch signaling in bone homeostasis. Nat. Med..

[22] Tao J., Chen S., Yang T., Dawson B., Munivez E., Bertin T., Lee B. (2010). Osteosclerosis owing to Notch gain of function is solely Rbpj-dependent. J. Bone Miner. Res..

[23] Yuan H., Wu S.X., Zhou Y.F., Peng F. (2021). Spermidine inhibits joints inflammation and macrophage activation in mice with collagen-induced arthritis. J. Inflamm. Res..

