# Supplementary material for: ADAM-10 Is Involved in Osteoblast Mineralization and Rheumatoid Arthritis-Associated Bone Destruction
Source: Int J Mol Sci. 2026 Nov 3;27(21):9842. doi: 10.3390/ijms27219842 (PMC13763782; doi:10.3390/ijms27219842)
Supplement: Supplementary file 1 [file ijms-27-09842-s001.zip › Table S1-HN.pdf]

Stage/Assessment areas	Number of patients
**Stage Ⅰ**	**8**
Wrists and ankle joints	8
**Stage Ⅱ**	**8**
Wrists	3
Left wrist	2
PIP joint of the ring finger, left fifth MTP joint	1
Right shoulder, left fifth MTP joint	1
Knees	1
**Stage Ⅲ**	**10**
Wrists	1
Left wrist	2
Left wrist and knees	1
MTP joints of both feet	2
MCP joints of the middle fingers	1
Left ring finger PIP joint	1
Left wrist, MTP joints	1
Wrists, elbows, and the MTP joint of the first toe	1
**Stage Ⅳ**	**4**
Wrists	1
Left Wrist	1
Wrists and knees	1
Wrists and MTP joints of both feet	1

Table S1. Details of the joints used in the Steinblocker classification. The number of patients per site is listed by stage.

Abbreviations: PIP, Proximal Interphalangeal joint; MTP, Metatarsophalangeal joint; MTP, Metacarpal phalangeal joint;
