# Supplementary material for: ADAM-10 Is Involved in Osteoblast Mineralization and Rheumatoid Arthritis-Associated Bone Destruction
Source: Int J Mol Sci. 2026 Nov 3;27(21):9842. doi: 10.3390/ijms27219842 (PMC13763782; doi:10.3390/ijms27219842)
Supplement: Supplementary file 1 [file ijms-27-09842-s001.zip › Table S2-HN.pdf]

Items	ADAM-10 Low group (n=3)	ADAM-10 High group (n=7)	p-value	FDR p-value
Age, years	74.7	78.1	0.58	0.81
Sex, female, n (%)	3(100)	4(57.1)	0.1	0.81
Height, cm	148.8	154.4	0.34	0.81
Weight, kg	48.0	47.5	0.9	0.98
BMI, kg/m^2	21.7	19.9	0.24	0.81
WBC, ×10^3/μL	8.3	7.2	0.67	0.84
Neut, /μL	6283.3	5307.1	0.66	0.84
Lym, /μL	1440	1364.3	0.83	0.96
ESR 1h, mm/hr	55	65	0.33	0.81
TP, g/dL	7.3	6.9	0.39	0.81
Alb, g/dL	3.1	2.8	0.52	0.81
BUN, mg/dL	15.7	32.7	0.19	0.81
Cr, mg/dL	0.4	1.1	0.3	0.81
eGFR, ml/min/1.73 ㎡	104.4	78.0	0.5	0.81
LDH, U/L	179.3	173.1	0.81	0.96
ALP, U/L	229.3	228.3	0.98	0.99
CRP, mg/dL	7.0	10.9	0.55	0.81
RF, IU/㎖	32.7	180.4	0.36	0.81
MMP-3, ng/ml	879.0	400.9	0.037	0.44
ACPA, U/ml	349.2	109.1	0.35	0.81

Table S2. Comparison of clinical characteristics and laboratory values between patients with high and low serum ADAM-10 concentrations in patients with Steinblocker Stage III. Patients were compared between the low ADAM-10 group (n = 3) and the high ADAM-10 group (n = 7). Student's t-test was used for comparisons between groups. P-values were corrected for multiple comparisons using the Benjamin-Hochberg method and are reported as false discovery rate (FDR) P-values. There were no items for which a statistically significant difference was observed in the FDR p-value ($p<0.05$).

Abbreviations: BMI, body mass index; WBC, white blood cell; Neut, neutrophil; Lym, lymphocyte; ESR 1h, erythrocyte sedimentation rate at 1 hour; TP, total protein; Alb, albumin; BUN, blood urea nitrogen; Cr, creatinine; eGFR, estimated glomerular filtration rate; LDH, lactate dehydrogenase; ALP, alkaline phosphatase; CRP, c-reactive protein; RF, rheumatoid factor; MMP-3, matrix metalloproteinase-3; ACPA, anti-citruillinated peptide antibodies; ADAM-10, a disintegrin and metalloprotease 10; FDR, false discovery rate.
